# A Constitutive Model for Soft Clays Incorporating Elastic and Plastic Cross-Anisotropy

**DOI:** 10.3390/ma10060584

**Published:** 2017-05-25

**Authors:** Jorge Castro, Nallathamby Sivasithamparam

**Affiliations:** 1 Department of Ground Engineering and Materials Science, University of Cantabria, Avda. de los Castros s/n, 39005 Santander, Spain; 2 Computational Geomechanics Division, Norwegian Geotechnical Institute (NGI), P.O. Box 3930, Ullevål Stadion, 0806 Oslo, Norway; nallathamby.siva@ngi.no

**Keywords:** natural structured clays, cross-anisotropy, constitutive model, anisotropy evolution, stress-dependent stiffness, triaxial tests

## Abstract

Natural clays exhibit a significant degree of anisotropy in their fabric, which initially is derived from the shape of the clay platelets, deposition process and one-dimensional consolidation. Various authors have proposed anisotropic elastoplastic models involving an inclined yield surface to reproduce anisotropic behavior of plastic nature. This paper presents a novel constitutive model for soft structured clays that includes anisotropic behavior both of elastic and plastic nature. The new model incorporates stress-dependent cross-anisotropic elastic behavior within the yield surface using three independent elastic parameters because natural clays exhibit cross-anisotropic (or transversely isotropic) behavior after deposition and consolidation. Thus, the model only incorporates an additional variable with a clear physical meaning, namely the ratio between horizontal and vertical stiffnesses, which can be analytically obtained from conventional laboratory tests. The model does not consider evolution of elastic anisotropy, but laboratory results show that large strains are necessary to cause noticeable changes in elastic anisotropic behavior. The model is able to capture initial non-vertical effective stress paths for undrained triaxial tests and to predict deviatoric strains during isotropic loading or unloading.

## 1. Introduction

The stress–strain response of natural soft clays is influenced by geological processes that occurred long time ago and consequently their response is much more complicated and unpredictable than that of any other man-made construction material. Natural soft clays exhibit a significant degree of anisotropy in their fabric, which initially is derived from the shape of the clay platelets, deposition process and one-dimensional consolidation. Neglecting this anisotropy of natural clay behavior may lead to highly inaccurate predictions of material response under loading [1].

Extensive experimental testing of soils under different stress paths and conditions as well as the increase in computing power has led to the development of advanced constitutive models that reproduce more accurately the mechanical behavior of soils. Some of these advanced constitutive models incorporate plastic anisotropy using a rotated and distorted elliptical yield surface [2,3,4,5]. These models assume elastic behavior is isotropic, yet it is known most natural clays will exhibit anisotropy of elastic behavior [6]. The reasons to avoid incorporating elastic anisotropy are usually the following [3]:

- To keep the model simple.
- Because plastic deformations are likely to dominate for many problems of practical interest.
- Because, if the model attempts to account for the development or erasure of anisotropy with subsequent loading, that leads to changes in the initial cross-anisotropic behavior, potentially resulting in fully generalized elastic anisotropy, involving 21 independent elastic parameters, which is clearly not practical.

Although the three above-mentioned reasons are sensible and justified, incorporating elastic cross-anisotropy to a model of this type is still useful because the model will better capture the elastic anisotropic behavior [7]. Besides, the development or erasure of elastic anisotropy requires important plastic strains; contrary to plastic anisotropy, which evolves at a much faster rate. For example, Mitchel [8] and Huekel and Tutumluer [9] showed that the rate of plastic anistropy demise is notably greater than that of elastic anisotropy after isotropic loading and unloading.

The MELANIE model [7] is a constitutive model for soft soils that incorporates both elastic and plastic anisotropy and has been used to model real boundary value problems, namely several experimental embankments in Cubzac-les-Ponts (France) [7]. However, the MELANIE model does not consider two important features of soft clay behavior: evolution of plastic anisotropy with plastic straining and stress-dependent elastic stiffness.

In this paper, an existing elastoplastic model that considers plastic anisotropy and its evolution with plastic straining, namely S-CLAY1S model [10], is extended to incorporate elastic cross-anisotropy, also known as transverse isotropy. To keep the model simple and for practical usage, only an additional parameter has been added to describe the elastic stress-dependent cross-anisotropy. Furthermore, evolution of elastic anisotropy is neglected. The additional elastic parameter has a clear physical meaning, namely the ratio between the horizontal and vertical stiffnesses, and it may be obtained analytically from a conventional triaxial test. The description of elastic cross-anisotropy is based on the three-parameter model proposed by Graham and Houlsby [6] for linear elasticity, but here, the stress-dependent response is also included.

The paper first presents the S-CLAY1S model (Section 2), as the proposed constitutive model is based on that model and the formulation of the plastic part is exactly the same. Next, the description of the elastic cross-anisotropy considered in the new constitutive model is developed (Section 3). Validation of the model using laboratory tests available in the literature and examples of parameter determination are presented in Section 4, and subsequently parametric studies are depicted in Section 5. Finally, some conclusions are derived.

The standard soil mechanics sign convention of compressive stresses and strains as positive is assumed, the prime notation is used to denote effective stresses ($\sigma^{\prime }$), and engineering shear strains are adopted ($\gamma_{ij}$). The stress and strain invariants are

(1) $$p^{\prime }=\left({\sigma^{\prime }}_{xx}\;+\;{\sigma^{\prime }}_{yy}\;+\;{\sigma^{\prime }}_{zz}\right)/3$$

(2) $$q\;=\;\sqrt{\frac{{\left({\sigma^{\prime }}_{xx}\;-\;{\sigma^{\prime }}_{yy}\right)}^{2}\;+\;{\left({\sigma^{\prime }}_{yy}\;-\;{\sigma^{\prime }}_{zz}\right)}^{2}\;+\;{\left({\sigma^{\prime }}_{xx}\;-\;{\sigma^{\prime }}_{zz}\right)}^{2}}{2}\;+\;3\left(\tau_{xy}^{2}\;+\;\tau_{yz}^{2}\;+\;\tau_{xz}^{2}\right)}$$

(3) $$\varepsilon_{v}\;=\;\varepsilon_{xx}\;+\;\varepsilon_{yy}\;+\;\varepsilon_{zz}$$

(4) $$\varepsilon_{d}\;=\;\frac{1}{3}\sqrt{2\left[{\left(\varepsilon_{xx}\;-\;\varepsilon_{yy}\right)}^{2}\;+\;{\left(\varepsilon_{yy}\;-\;\varepsilon_{zz}\right)}^{2}{\left(\varepsilon_{xx}\;-\;\varepsilon_{zz}\right)}^{2}\right]\;+\;3\left(\gamma_{xy}^{2}\;+\;\gamma_{xz}^{2}\;+\;\gamma_{yz}^{2}\right)}$$

which, for triaxial conditions, reduce to

(5) $$p^{\prime }\;=\;\left({\sigma^{\prime }}_{a}\;+\;2{\sigma^{\prime }}_{r}\right)/3$$

(6) $$q\;=\;{\sigma^{\prime }}_{a}\;-\;{\sigma^{\prime }}_{r}$$

(7) $$\varepsilon_{v}\;=\;\varepsilon_{a}\;+\;2\varepsilon_{r}$$

(8) $$\varepsilon_{d}\;=\;2\left(\varepsilon_{a}\;-\;\varepsilon_{r}\right)/3$$

*x*,*y*,*z* denote rectangular Cartesian coordinates. For triaxial conditions, *a* and *r* subscripts indicate axial and radial principal directions, respectively.

## 2. S-CLAY1S Model

**Figure 1:**
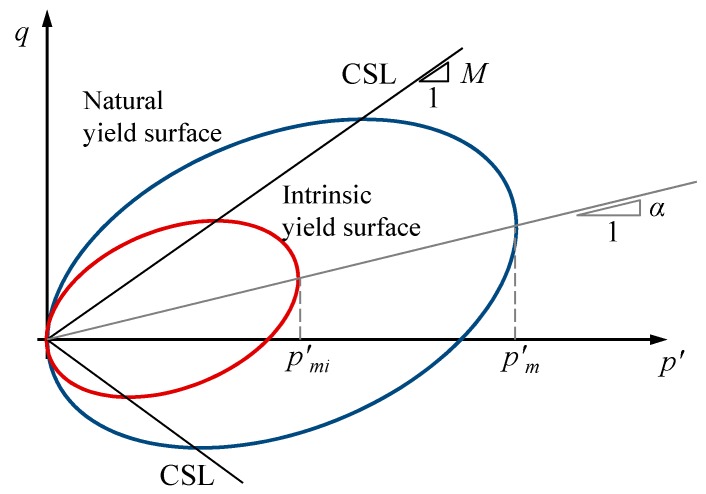
Yield surfaces of the S-CLAY1S model (Modified from [10]).

S-CLAY1S [10] is a Modified Cam Clay (MCC) [11] type model that accounts for plastic anisotropy and destructuration. Anisotropy of plastic behavior is represented through an inclined and distorted elliptical yield surface and a rotational hardening law to model the development or erasure of fabric anisotropy during plastic straining, while interparticle bonding and degradation of bonds (structure) is reproduced using intrinsic and natural yield surfaces [12] and a hardening law describing destructuration as a function of plastic straining. The last letter of the model (“S”) refers to the soil structure. Thus, when the hierarchical version of the model without destructuration is used, the model is simply called S-CLAY1 [3]. For the sake of simplicity, the mathematical formulation is presented in the following in triaxial stress space, which can be used only to model the response of cross-anisotropic samples (cut vertically from the soil deposit) subject to oedometer or triaxial loading. The original inclined yield surface of the S-CLAY1 model is elliptical [3] (see Figure 1):

(9) $$f_{y}\;=\;1\;+\;\frac{{\left(\eta \;-\;\alpha \right)}^{2}}{M^{2}\;-\;\alpha^{2}}\;-\;\frac{p_{mi}^{\prime }}{p^{\prime }}\;=\;0$$

where *η* is the stress ratio, *M* is the stress ratio at critical state, $p^{\prime }$ is the mean effective stress, $p_{mi}^{\prime }$ is the size of the intrinsic yield surface related to the soil’s pre-consolidation pressure and *α* is the plastic anisotropy parameter that gives the inclination of the yield surface. In triaxial stress space, *α* is a scalar value, but, in the full three-dimensional formulation, **α** is a fabric tensor (3 × 3).

The effect of bonding in the S-CLAY1S model [10] is described by an intrinsic yield surface [12] that has the same shape and inclination of the natural yield surface but with a smaller size (see Figure 1). The size of the intrinsic yield surface is specified by parameter $p_{mi}^{\prime }$ which is related to the size $p_{m}^{\prime }$ of the natural yield surface by parameter χ as the current amount of bonding

(10) $$p_{m}^{\prime }=\left(1+\chi \right)p_{mi}^{\prime }$$

S-CLAY1S model incorporates three hardening laws. The first of these is the isotropic hardening law similar to that of MCC model [11] that controls the expansion or contraction of the intrinsic yield surface as a function of the increments of plastic volumetric strains ($d\varepsilon_{v}^{p}$)

(11) $$dp_{mi}^{\prime }\;=\;\frac{vp_{mi}^{\prime }}{\lambda_{i}\;-\;\kappa }d\varepsilon_{v}^{p}$$

where v is the specific volume, $\lambda_{i}$ is the gradient of the intrinsic normal compression line in the compression plane ($\ln p^{\prime }\;-\;v$ space), κ is the slope of the swelling line in the compression plane, and $d\varepsilon_{v}^{p}$ is the increment of plastic volumetric strain. The second hardening law is the rotational hardening law, which describes the rotation of the yield surface with plastic straining [3]

(12) $$d\alpha =\omega \left[\left(\frac{3\eta }{4}\;-\;\alpha_{d}\right)\left\langle d\varepsilon_{v}^{p}\right\rangle \;+\;\omega_{d}\left(\frac{\eta }{3}\;-\;\alpha_{d}\right)\left|d\varepsilon_{d}^{p}\right|\right]$$

where $d\varepsilon_{d}^{p}$ is the increment of plastic deviatoric strain, and ω and $\omega_{d}$ are additional soil constants that control, respectively, the absolute rate of rotation of the yield surface toward its current target value, and the relative effectiveness of plastic deviatoric strains and plastic volumetric strains in rotating the yield surface. The third hardening law in S-CLAY1S model is for destructuration, which describes the degradation of bonding with plastic straining. The destructuration law is formulated in such a way that both plastic volumetric strains and plastic shear strains tend to decrease the value of the bonding parameter χ towards a target value of zero [10], it is defined as

(13) $$d\chi \;=\;-\xi \chi \left(\left|d\varepsilon_{v}^{p}\right|\;+\;\xi_{d}\left|d\varepsilon_{d}^{p}\right|\right)$$

where ξ and $\xi_{d}$ are additional soil constants. Parameter ξ controls the absolute rate of destructuration, and parameter $\xi_{d}$ controls the relative effectiveness of plastic deviatoric strains and plastic volumetric strains in destroying the inter-particle bonding [13].

The elastic behavior in the model is formulated as isotropic, with elastic increments of volumetric and deviatoric strains given by the same expressions as in MCC:

(14) $$\left[\begin{array}{c} d\varepsilon_{v}^{e}\\ d\varepsilon_{d}^{e} \end{array}\right]\;=\;\left[\begin{array}{cc} 1/K & 0\\ 0 & 1/3G \end{array}\right]\left[\begin{array}{c} dp^{\prime }\\ dq \end{array}\right]$$

where K is a stress-dependent elastic bulk modulus ($K=\nu p^{\prime }/\kappa$). The model has traditionally been implemented assuming a constant Poisson’s ratio, v [3,14]. Thus, the shear modulus G is related to the elastic bulk modulus K, and is also stress dependent, instead of being constant.

S-CLAY1S has proven its ability to reproduce the behavior of lightly overconsolidated structured soft clays both for laboratory tests and for boundary value problems [15,16,17]. The main advantages of the S-CLAY1S model are: (i) its relatively simple model formulation; (ii) its realistic $K_{0}$ prediction; and, most importantly; (iii) the fact that model parameter values can be determined from standard laboratory tests using well-defined methodologies [10]. S-CLAY1S was chosen to keep the model simple, but there are more advanced constitutive models for soft clays that, for example, introduce some flexibility in the shape of the plastic potential [14] or use a non-associated flow rule [18,19]. Some other advanced features of clay behavior, such as creep [20] or small-strains [21], have not been here considered either.

## 3. Stress-Dependent Elastic Cross-Anisotropy

### 3.1. Linear Elastic Cross-Anisotropy

Fully generalized elastic anisotropy involves 21 independent elastic parameters due to the symmetry of the 6 × 6 stiffness matrix that relates the six independent stress increment components with the six independent strain increment components. The elastic matrix should be symmetric to satisfy thermodynamic principles [22,23]. However, most materials present some kind of symmetry that allows reducing the number of independent elastic parameters. Soil properties are influenced by their deposition and stress history, which are controlled by the gravity acceleration acting on the vertical axis (deposition and one-dimensional consolidation). Thus, soft clays that have not undergone any other types of straining, such as those caused by tectonic movements, present a cross-anisotropic or transversely isotropic behavior, i.e., both horizontal axes are interchangeable. That reduces the number of independent elastic parameters to 5, which are often chosen as *E_V_*, *E_H_*, *ν_VV_*, *ν_VH_* and *G_VH_*, where *V* and *H* subscripts refer to vertical and horizontal directions, respectively. The compliance matrix that relates the stress increments and the strain increments (inverse of the stiffness matrix) is as follow

(15) $$\left[\begin{array}{c} d\varepsilon_{xx}^{e}\\ d\varepsilon_{yy}^{e}\\ d\varepsilon_{zz}^{e}\\ d\gamma_{xy}^{e}\\ d\gamma_{yz}^{e}\\ d\gamma_{zx}^{e} \end{array}\right]=\;\left[\begin{array}{cccccc} 1/E_{H} & -\nu_{HH}/E_{H}\; & -\nu_{HV}/E_{V} & 0 & 0 & 0\\ -\nu_{HH}/E_{H}\; & 1/E_{H} & -\nu_{HV}/E_{V}\; & 0 & 0 & 0\\ -\nu_{HV}/E_{V}\; & -\nu_{HV}/E_{V}\; & 1/E_{V} & 0 & 0 & 0\\ 0 & 0 & 0 & 1/G_{HV} & 0 & 0\\ 0 & 0 & 0 & 0 & 1/G_{HV} & 0\\ 0 & 0 & 0 & 0 & 0 & 2\left(1+\nu_{HH}\right)/E_{H} \end{array}\right]\left[\begin{array}{c} d{\sigma^{\prime }}_{xx}\\ d{\sigma^{\prime }}_{yy}\\ d{\sigma^{\prime }}_{zz}\\ d\tau_{xy}\\ d\tau_{yz}\\ d\tau_{zx} \end{array}\right]$$

Rectangular Cartesian axes (*x*, *y*, *z*) are here used and *z* is taken as the vertical direction. While the vertical and horizontal stiffnesses (*E_V_*, *E_H_*) have a clear physical meaning, distinguishing between different Poisson’s ratios and adding an independent shear modulus adds complexity to the study.

Graham and Houlsby [6] showed that only three parameters can be deduced from triaxial tests on vertically cut specimens and proposed a simplified three parameter model (E*, ν*, *α_e_*). This is also called one-parameter anisotropy because only one parameter is added to describe anisotropy. Here, the subscript “*e*” is introduced in the *α* parameter to indicate that it refers to the elastic part and to distinguish it from the *α* parameter of the S-CLAY1S model that controls anisotropy in the plastic part. The stiffness matrix of this model is

(16) $$\left[\begin{array}{c} d{\sigma^{\prime }}_{xx}\\ d{\sigma^{\prime }}_{yy}\\ d{\sigma^{\prime }}_{zz}\\ d\tau_{xy}\\ d\tau_{yz}\\ d\tau_{zx} \end{array}\right]=\frac{E^{*}}{\left(1\;+\;\nu^{*}\right)\left(1\;-\;2\nu^{*}\right)}[\begin{array}{cccccc} \alpha_{e}^{2}\left(1\;-\;\nu^{*}\right) & \alpha_{e}^{2}\nu^{*} & \alpha_{e}\nu^{*} & 0 & 0 & 0\\ \alpha_{e}^{2}\nu^{*} & \alpha_{e}^{2}\left(1\;-\;\nu^{*}\right) & \alpha_{e}\nu^{*} & 0 & 0 & 0\\ \alpha_{e}\nu^{*} & \alpha_{e}\nu^{*} & \left(1\;-\;\nu^{*}\right) & 0 & 0 & 0\\ 0 & 0 & 0 & \frac{\alpha_{e}}{2}\left(1\;-\;2\nu^{*}\right) & 0 & 0\\ 0 & 0 & 0 & 0 & \frac{\alpha_{e}}{2}\left(1\;-\;2\nu^{*}\right) & 0\\ 0 & 0 & 0 & 0 & 0 & \frac{\alpha_{e}}{2}\left(1\;-\;2\nu^{*}\right) \end{array}]\left[\begin{array}{c} d\varepsilon_{xx}^{e}\\ d\varepsilon_{yy}^{e}\\ d\varepsilon_{zz}^{e}\\ d\gamma_{xy}^{e}\\ d\gamma_{yz}^{e}\\ d\gamma_{zx}^{e} \end{array}\right]$$

and the compliance matrix (inverse of Equation (16)) is

(17) $$\left[\begin{array}{c} d\varepsilon_{xx}^{e}\\ d\varepsilon_{yy}^{e}\\ d\varepsilon_{zz}^{e}\\ d\tau_{xy}\\ d\tau_{yz}\\ d\tau_{zx} \end{array}\right]\;=\frac{1}{E^{*}}\left[\begin{array}{cccccc} 1/\alpha_{e}^{2} & {-}^{\nu *}/\alpha_{e}^{2}\; & {-}^{\nu *}/\alpha_{e} & 0 & 0 & 0\\ {-}^{\nu *}/\alpha_{e}^{2}\; & 1/\alpha_{e}^{2} & {-}^{\nu *}/\alpha_{e}\; & 0 & 0 & 0\\ {-}^{\nu *}/\alpha_{e}\; & {-}^{\nu *}/\alpha_{e}\; & 1 & 0 & 0 & 0\\ 0 & 0 & 0 & 2\left(1\;+{\;}^{\nu *}\right)/\alpha_{e} & 0 & 0\\ 0 & 0 & 0 & 0 & 2\left(1\;+{\;}^{\nu *}\right)/\alpha_{e} & 0\\ 0 & 0 & 0 & 0 & 0 & 2\left(1\;+{\;}^{\nu *}\right)/\alpha_{e}^{2} \end{array}\right]\left[\begin{array}{c} d{\sigma^{\prime }}_{xx}\\ d{\sigma^{\prime }}_{yy}\\ d{\sigma^{\prime }}_{zz}\\ d\tau_{xy}\\ d\tau_{yz}\\ d\tau_{zx} \end{array}\right]$$

Comparing Equation (17) with Equation (15), it can be established that $E_{V}\;=\;E^{*}$, $E_{H}\;=\;\alpha_{e}^{2}E^{*}$, $\nu_{HH}\;=\;\nu^{*}$, $\nu_{HV}\;=\;\nu^{*}/\alpha_{e}$, and $2G_{HV}\;=\;\alpha_{e}E^{*}/\left(1\;+\;\nu^{*}\right)$.

### 3.2. Effects of Elastic Cross-Anisotropy in Triaxial Tests

For triaxial tests, Graham and Houlsby [6] showed that the complete stiffness matrix (Equation (16)) reduces to:

(18) $$\left[\begin{array}{c} dp^{\prime }\\ dq \end{array}\right]\;=\;\left[\begin{array}{cc} K^{*} & J\\ J & 3G^{*} \end{array}\right]\left[\begin{array}{c} d\varepsilon_{v}^{e}\\ d\varepsilon_{d}^{e} \end{array}\right]$$

where $K^{*}$ and $G^{*}$ are modified values of bulk modulus and shear modulus, and the off-diagonal term *J* shows that there is some cross-coupling between volumetric and distortional effects, contrary to what happens under isotropic elasticity (Equation (14)). Manipulating the stiffness matrix (Equation (16)), $K^{*}$, $G^{*}$ and J may be expresses in terms of E*, ν*, and *α_e_* [24] (p. 49):

(19) $$K^{*}=\frac{E^{*}\left(1-\nu^{*}+4\alpha_{e}\nu^{*}+2\alpha_{e}^{2}\right)}{9\left(1+\nu^{*}\right)\left(1-2\nu^{*}\right)}$$

(20) $$G^{*}=\frac{E^{*}\left(2-2\nu^{*}-4\alpha_{e}\nu^{*}+\alpha_{e}^{2}\right)}{6\left(1+\nu^{*}\right)\left(1-2\nu^{*}\right)}$$

(21) $$J=\frac{E^{*}\left(1-\nu^{*}+\alpha_{e}\nu^{*}-\alpha_{e}^{2}\right)}{3\left(1+\nu^{*}\right)\left(1-2\nu^{*}\right)}$$

The coupling between volumetric and distortional effects implies that:

- Under constant volume effective stress paths ($d\varepsilon_{v}^{e}=0$), the mean effective stress is no longer constant ($dp^{\prime }\neq 0$), i.e., the stress path in the *p’*:*q* plane is no longer vertical and depends on the value of $\alpha_{e}$ [6,24] as follows (using Equations (18), (20) and (21))
  (22) $$\frac{dq}{dp^{\prime }}\;=\;\frac{3G^{*}}{J}\;=\;\frac{3\left(2\;-\;2\nu^{*}\;-\;4\alpha_{e}\nu^{*}\;+\;\alpha_{e}^{2}\right)}{2\left(1\;-\;\nu^{*}\;+\;\alpha_{e}\nu^{*}\;-\;\alpha_{e}^{2}\right)}$$

- Under constant q stress paths (dq = 0), shear strains will occur ($d\varepsilon_{d}^{e}\;\neq \;0$). For example, for isotropic compression (q = 0), the ratio between shear and volumetric strains depends on the value of $\alpha_{e}$ [6,24] as follows (using Equations (18), (20) and (21))
  (23) $$\frac{d\varepsilon_{d}^{e}}{d\varepsilon_{v}^{e}}\;=\;\frac{-J}{3G^{*}}\;=\;\frac{-2\left(1\;-\;\nu^{*}\;+\;\alpha_{e}\nu^{*}\;-\;\alpha_{e}^{2}\right)}{3\left(2\;-\;2\nu^{*}\;-\;4\alpha_{e}\nu^{*}\;+\;\alpha_{e}^{2}\right)}$$

### 3.3. Proposed Stress-Dependend Cross-Anisotropy

The elastic cross-anisotropic model by Graham and Houlsby [6] has been chosen as the basis for the current stress-dependent model because it allows to keep $E^{*}$ and $\nu^{*}$ with a similar meaning and the same values as the traditional E and ν parameters. Besides, the only additional parameter (elastic anisotropic parameter, $\alpha_{e}$) has a clear physical meaning ($\alpha_{e}^{2}\;=\;E_{H}/E_{V}$) and may be analytically obtained from conventional triaxial test, either from the undrained stress path during shearing (Equation (22)) or from the shear-volumetric strain ratio during drained isotropic compression (Equation (23)). Graham and Houlsby [6] model cannot be regarded as a full constitutive model and is only used for a very limited range of strains in soft soils because it does not consider stress-dependent stiffness nor plastic strains.

The stress-dependent stiffness of the present model has been introduced relating the E* parameter with κ through a linear dependency on the effective mean pressure $p^{\prime }$. To preserve the original meaning of κ as the slope of the swelling line in the compression plane ($\ln p^{\prime }\;-\;v$ space), the corresponding one-dimensional (confined) compression modulus has been used ($dp^{\prime }/d\epsilon_{zz})$, which can be obtained from Equation (16) taking ($d\epsilon_{xx}\;=\;d\epsilon_{yy}\;=\;d\epsilon_{ij}\;=\;0)$

(24) $$\frac{dp^{\prime }}{d\varepsilon_{zz}^{e}}\;=\;\frac{E^{*}\left(1\;-{\;}^{\nu *}\;+\;2\alpha_{e}\nu^{*}\right)}{3\left(1\;+\nu^{*}\right)\left(1\;-\;2\nu^{*}\right)}$$

For isotropic elasticity ($\alpha_{e}\;=\;1$), Equation (24) reduces to the bulk modulus K

(25) $$\frac{dp^{\prime }}{d\varepsilon_{zz}^{e}}\;=\;\frac{E}{3\left(1\;-\;2\nu \right)}\;=\;K$$

The stress-dependent stiffness of a soil under unloading or reloading in the compression plane is given by

(26) $$\frac{de^{e}}{dp^{\prime }}\;=\;-\frac{\kappa }{p^{\prime }}$$

where the variation of the void ratio that corresponds to the elastic part ($de^{e}$) may be related to the increment of the vertical elastic strain $d\varepsilon_{zz}^{e}$

(27) $$d\varepsilon_{zz}^{e}\;=\;-\frac{de^{e}}{1\;+\;e}$$

Thus, using Equations (24), (26) and (27), the stress-dependent expression for the E* parameter is obtained

(28) $$E^{*}=\frac{3\left(1\;+\nu^{*}\right)\left(1\;-\;2\nu^{*}\right)}{1\;-{\;}^{\nu *}\;+\;2\alpha_{e}\nu^{*}}\frac{\left(1\;+\;e\right)}{\kappa }p^{\prime }$$

The stress-dependent elastic cross-anisotropy used in the present model is given by Equations (17) and (28). The present formulation of elastic anisotropy is hierarchical because if $\alpha_{e}\;=\;1$, the model reduces to S-CLAY1S [10] with isotropic elasticity. The current formulation of elasticity may be considered as hypoelastic [25], as it is defined in terms of stress and strain increments (Equation (17)) The authors are aware that this model is non-conservative [26], but assuming a constant Poisson’s ratio is widely used [3] and the differences for non-cyclic loads are small for practical purposes [27]. Application of a hyperelastic formulation [28] can solve the problem. However, most hyperelastic models [28,29] are fundamentally isotropic and only predict some stress-induced coupling between volumetric and deviatoric parts [30] (p. 151). A general stress formulation [30] (p. 153) may be used to model stress-induced anisotropy. Both stress-induced coupling [28,29] and stress-induced anisotropy predict an isotropic elastic response when the stress state is at the isotropic compression line. To properly predict strain-induced anisotropic elasticity within a hyperelastic formulation, a coupled elastic-plastic formulation is required, i.e., the energy potential should depend on the plastic strains [9], and that leads to a highly complex model.

On the other hand, it is worth noting that results of oedometer tests are often plotted using a decimal logarithm scale (i.e., with base 10) for the vertical stress on the horizontal axis. For the virgin compression line, the relationship between the compression index $C_{c}$ and λ is exact ($C_{c}\;=\;\lambda \cdot log_{10}e$). However, for the unloading-reloading path, there is not an exact relationship between the swelling index $C_{s}$ and κ, because the ratio of horizontal and vertical stresses changes during one-dimensional unloading. If the ratio of horizontal and vertical stresses were the same as for the virgin compression line, the relationship would be $C_{s}\;\approx \;\kappa \cdot log_{10}e$. However, it is more realistic to assume that this ratio increases, and if an average value of 1 is adopted, i.e., horizontal stress approximately equal to vertical stress, the relationship is $C_{s}\;\approx \;\kappa \cdot \left(log_{10}e\right)/2$.

## 4. Validation

The validation aims to shown the capabilities of the proposed model, which includes anisotropic elasticity in a simple way. The results predicted by the model are compared with laboratory tests on a well-documented soft natural clay, namely Bothkennar clay [31,32,33]. The results of the proposed model are compared with the results of S-CLAY1 ($\alpha_{e}\;=\;1$) to highlight the influence of anisotropic elasticity. Parameter calibration for S-CLAY1 is based on the method proposed by Wheeler et al. [3]. The additional parameter of the proposed model ($\alpha_{e}$) is estimated using Equations (22) or (23). To simplify the validation, soil destructuration has been initially neglected (χ = 0), i.e., S-CLAY1 instead of S-CLAY1S.

**Figure 2:**
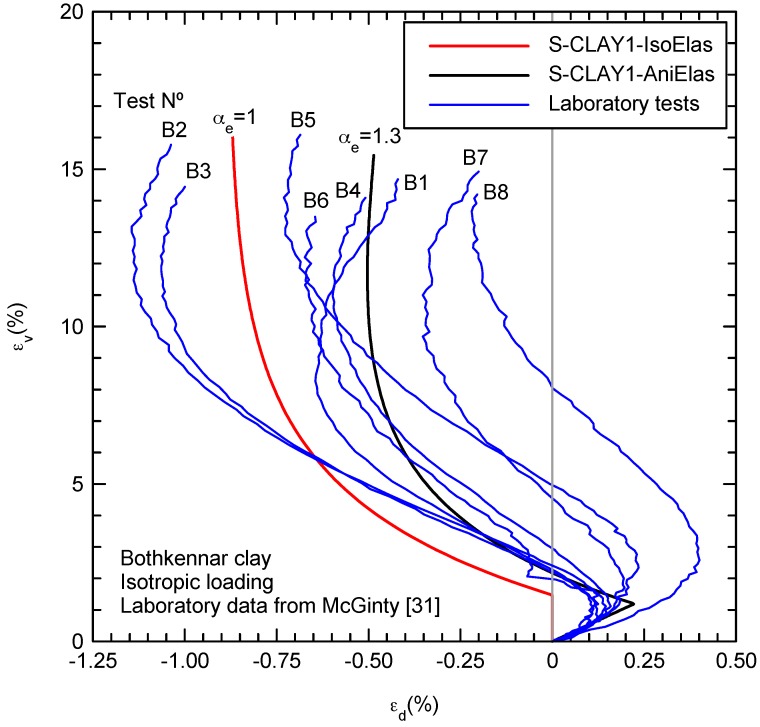
Influence of elastic anisotropy during isotropic compression (thin blue lines are laboratory results [31] and thicker red and black lines are S-CLAY1 predictions using isotropic and anisotropic elasticity, respectively).

The Bothkennar soft clay test site is located on the south side of the Firth of Forth, in Scotland, and has been the subject of a number of comprehensive studies [31,32,33]. The Bothkennar clay is a dark grey/black micaceus clayey silt, in part thinly laminated and mottled, that was deposited in a stable marine environment. Typically, in a structured soil, the in situ water content is close to the liquid limit. The Bothkennar clay is remarkably uniform in composition and mineralogy but exhibits natural variability in structure, fabric and state. It has a sand content of less than 10% and a clay fraction generally of around 30% [32]. The principal clay mineral is illite, but the plasticity (plasticity index in the range 25–55%) appears enhanced by organic residues (3–8%), mainly from marine organisms. The mineral assemblage is predominantly quartz and feldspar flour, kaolinite and illite/mica [33]. The sensitivity varies with the facies type, being around 5–8 in the mottled facies and between 7 and 13 in the bedded and laminated facies [33]. Table 1 shows the adopted parameters for the proposed model. $e_{0}\;$ is the initial void ratio and ${\sigma^{\prime }}_{Vc}$ is the preconsolidation stress. The additional parameter of the proposed model ($\alpha_{e}\;=\;1.3$) was analytically obtained using Equation (23) from the ratio of deviatoric to volumetric strain increments ($d\varepsilon_{d}^{e}/d\varepsilon_{v}^{e}\;=\;0.2$) measured in the laboratory [31] during drained isotropic compression at pressures lower than the preconsolidation one, i.e., initial straight part in Figure 2. $\alpha_{e}\;=\;1.3$ means that the ratio of horizontal to vertical elastic stiffness is equal to $E_{H}/E_{V}\;=\;1.7$. Those values are rounded to one decimal due to the lack of higher precision.

**Figure 3:**
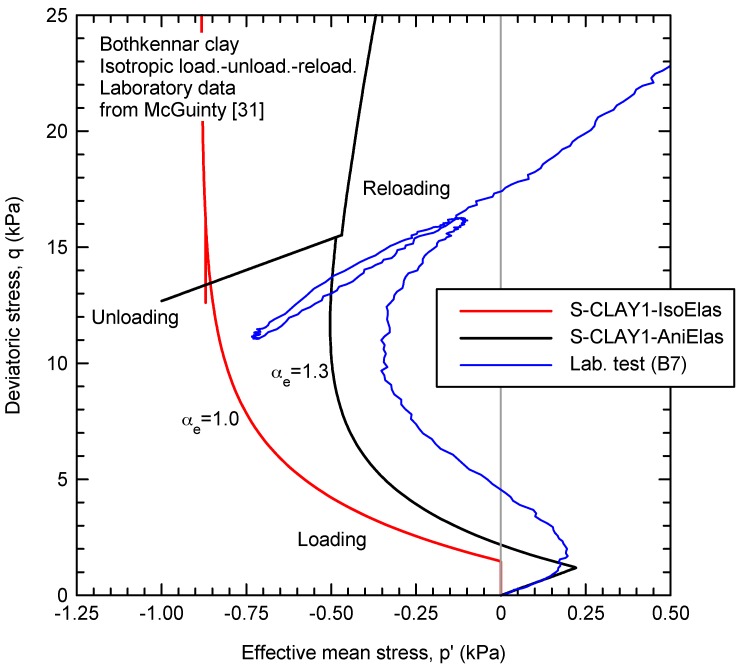
Influence of elastic anisotropy for an isotropic unloading-reloading loop.

Figure 2 shows how the proposed model reproduces the deviatoric strains during isotropic compression. Initially, during the elastic part, positive deviatoric strains ($d\varepsilon_{d}^{e}\;>\;0$) are predicted because the higher horizontal elastic stiffness ($\alpha_{e}\;>\;1$). After yielding, the deviatoric strain increments are mainly plastic and negative ($d\varepsilon_{d}^{p}\;<\;0$). Negative plastic deviatoric strain increments are predicted by the model because the yield surface is rotated and an associated plastic flow rule is adopted (see Figure 1). Obviously, yielding is sharply represented by the constitutive model, while the laboratory results [31] show a smooth transition. As plastic strains develop during isotropic loading, plastic anisotropy demises, the yield surface rotates toward the hydrostatic axis and plastic deviatoric strains tend to be 0 ($d\varepsilon_{d}^{p}\;=\;0$). If elastic anisotropy is considered, deviatoric strains tend to be positive again as the laboratory results show for the final part (Figure 2). This is clearer if an unloading-reloading loop is performed at the end of the test as done in test B7 by McGinty [31] (Figure 3). The constitutive model with isotropic elasticity predicts a vertical straight line ($d\varepsilon_{d}^{e}\;=\;0$) for the unloading-reloading loop, while an inclined straight line is predicted when anisotropic elasticity is included. The strain path direction is the same as that at the beginning of the test ($d\varepsilon_{d}^{e}/d\varepsilon_{v}^{e}\;=\;0.2$) for the proposed constitutive model because a constant elastic anisotropy is adopted, while McGinty [31] data show a demise of elastic anisotropy ($d\varepsilon_{d}^{e}/d\varepsilon_{v}^{e}\;=\;0.12$). An anomaly in McGinty [31] data is that no detectable change in strain path direction associated with the yield point is observed after reloading. This anomaly may be attributed to testing imperfections at high strains (>15%).

**Figure 4:**
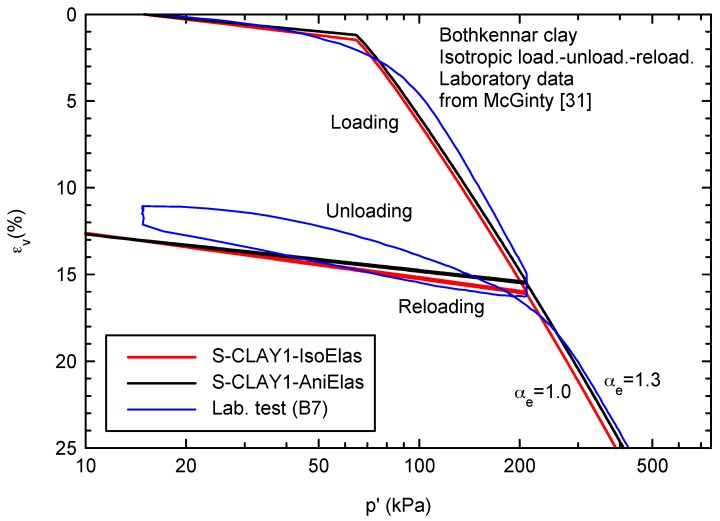
Stress–strain behavior during isotropic loading.

For drained isotropic loading, the study has focused on the strain paths because the differences in the stress–strain behavior are small (Figure 4). The results are slightly different because the elastic bulk modulus slightly depends on $\alpha_{e}$ (Equations (19) and (28)).

**Figure 5:**
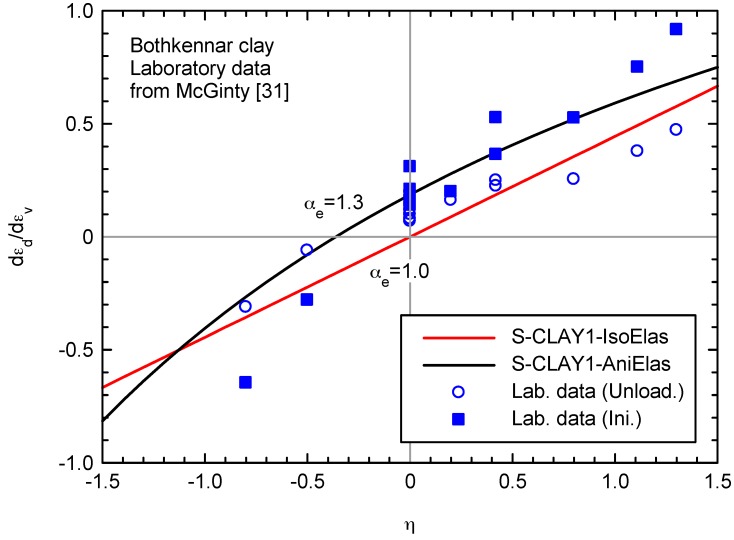
Variation of strain path direction with stress ratio (Ini.: initial loading; Unload.: unloading).

McGinty [31] performed drained triaxial tests at different stress ratios (see Table 2). The ratio of deviatoric to volumetric strain increments ($d\varepsilon_{d}^{e}/d\varepsilon_{v}^{e}$) was approximately determined from both the initial elastic part and from the unloading stage for isotropic compression (Table 3) and for different stress ratios (Table 2). Although the determination for the initial elastic part is difficult as plastic strains quickly appear and it is difficult to ensure a true cut off point between pre-yield and post-yield behavior, Table 2 and Table 3 show a demise in the elastic anisotropy, i.e., $d\varepsilon_{d}^{e}/d\varepsilon_{v}^{e}$ for the unloading phase is roughly half of the initial value. The results are summarized in Figure 5.

In the proposed model, the $d\varepsilon_{d}^{e}/d\varepsilon_{v}^{e}$ ratio depends on the stress ratio (η) as follows:

(29) $$\frac{d\varepsilon_{d}^{e}}{d\varepsilon_{v}^{e}}\;=\;\frac{K^{*}\eta \;-\;J}{3G^{*}\;-\;J\eta }$$

For the isotropic case ($\alpha_{e}\;=\;1$), the $d\varepsilon_{d}^{e}/d\varepsilon_{v}^{e}$ ratio linearly varies with the stress ratio (η):

(30) $$\frac{d\varepsilon_{d}^{e}}{d\varepsilon_{v}^{e}}\;=\;\frac{K}{3G}\eta \;=\;\frac{2\left(1\;+\;\nu \right)}{9\left(1\;-\;2\nu \right)}\eta$$

**Figure 6:**
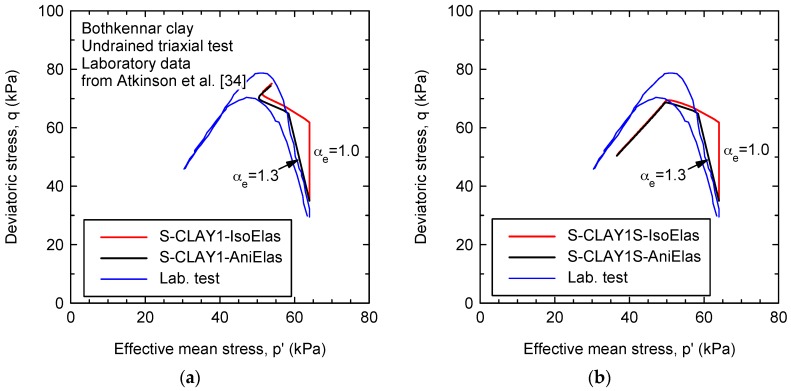
Effective stress path for undrained triaxial compression: (**a**) simulations using S-CLAY1; and (**b**) simulations using S-CLAY1S (considering destructuration).

Atkinson et al. [34] present results of undrained triaxial compression tests carried out on samples of intact Bothkennar clay recovered using a Laval sampler. The laboratory test samples were cut from the Laval sample using different techniques. As shown in Figure 6, the effective stress path in the *p’*:*q* plane is no longer vertical for the initial overconsolidated (elastic) part due to the anisotropic elastic behavior. As previously mentioned, under constant volume effective stress paths ($d\varepsilon_{v}^{e}\;=\;0$), the mean effective stress is no longer constant ($dp^{\prime }\neq \;0$) and depends on the value of $\alpha_{e}$ (Equation (22)) for the proposed anisotropic model. The fitted value $\alpha_{e}\;=\;1.3$ analytically obtained using Equation (23) and McGinty [31] data agrees with that obtained using Equation (22) and Atkinson et al. [34] data.

The depth of the samples used by Atkinson et al. [30], namely 12.6 m, is slightly higher than those samples used by McGinty [31] (around 11 m depth). Thus, the parameters used for the simulations shown in Figure 6a are those in Table 1, but for the presconsolidation stress (${\sigma^{\prime }}_{Vc}\;$= 105 kPa). To improve the agreement between laboratory results and the proposed model, numerical simulations including destructuration (S-CLAY1S) are also depicted in Figure 6b. The parameters used to model destructuration are shown in Table 4. The initial elastic part is not affected when destructuration is included, but the final plastic part shows a notable reduction in the stress level along the critical state line in agreement with the laboratory tests.

Here validation limits to Bothkennar clay, but similar results would be obtained for other soft clays, for example, Winnipeg clay [6]. Winnipeg clay is a post-glacial plastic clay from Lake Agassiz, Canada. The clay-size fraction is often as much as 75–80%, while the clay minerals are mostly interlayered montmorillonite-illites [35]. Although both Bothkennar and Winnipeg clay are soft natural structured clays, most of their properties are different, e.g., the drained strength and plastic anisotropy [3]. The anisotropic elastic parameter slightly varies with the depth sample for Winnipeg clay andits value is around 1.2–1.4 [6]; thus, it is similar to that obtained for Bothkennar clay (1.3).

## 5. Parametric Analyses

**Figure 7:**
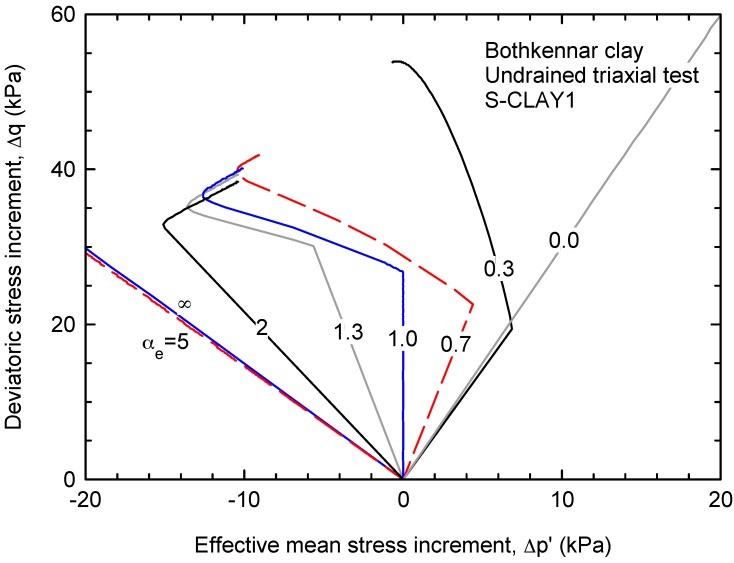
Effective stress path for undrained triaxial compression (each line corresponds to the anisotropic elastic parameter, $\alpha_{e}$, that is indicated over the line).

To highlight the influence of some of the model parameters, particularly the anisotropic elastic parameter $\alpha_{e}$, parametric analyses were performed. Figure 7 shows the increments of the effective stress paths for undrained triaxial compression tests. The results and parameters in Figure 7 are based on the undrained triaxial tests performed by [34] and presented in Figure 6. Since the focus is on the influence of the anisotropic elastic parameter on the initial slope of the effective stress path, the increments of deviatoric and mean stresses are presented in Figure 7. The slope of the effective stress ratio has limiting values of −1.5 and +3 for very large and very small values of $\alpha_{e}$, respectively.

**Figure 8:**
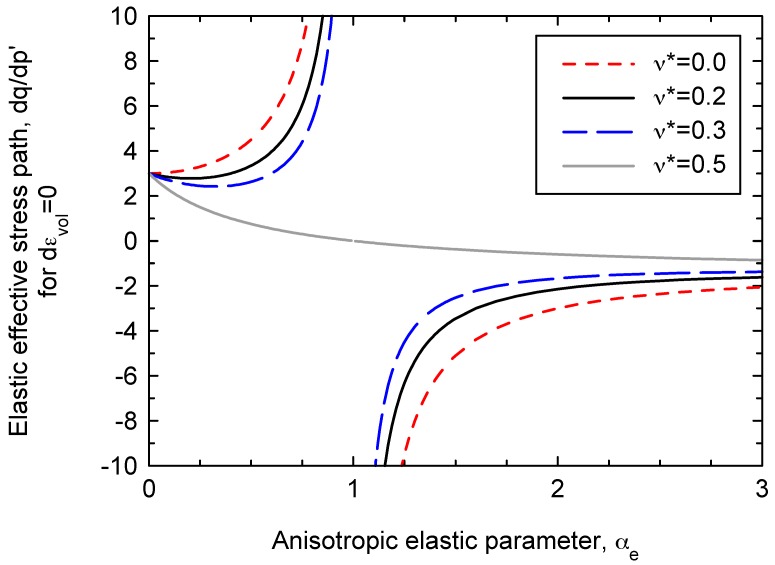
Elastic effective stress path for compression under constant volume ($d\varepsilon_{v}^{e}\;=\;0$)

The variation of the slope of the effective stress path with the anisotropic elastic parameter is given by Equation (22) and plotted in Figure 8 for several values of the Poisson’s ratio. The relationship is valid for the presented model and for the linear elastic model by Graham and Houlsby [6].

**Figure 9:**
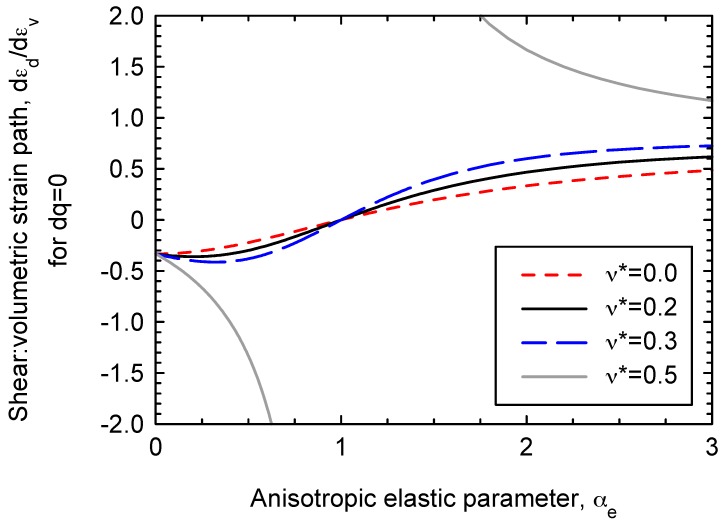
Strain path (ratio between shear and volumetric strains) for compression under constant shear stresses (dq = 0)

**Figure 10:**
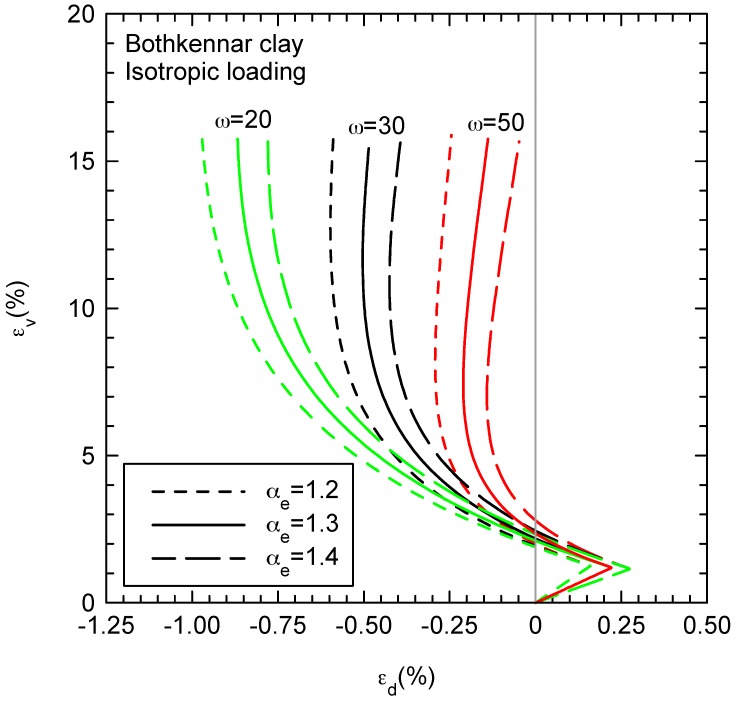
Strain path (ratio between shear and volumetric strains) for compression under constant shear stresses (dq = 0)

On the other hand, the slope of the strain paths (ratio between deviatoric and volumetric strains) for isotropic compression is given by Equation (23) for the presented model and for the linear elastic model by [6]. The relationship is plotted in Figure 9. The slope of the strain path has limiting values of 2/3 and −1/3 for very large and very small values of $\alpha_{e}$, respectively. Using the case presented in Figure 2 as a basis, a parametric analysis is presented in Figure 10. The anisotropic elastic parameter ($\alpha_{e}$) is slightly varied between 1.2 and 1.4. It is remarkable that $\alpha_{e}$ influences the slope of the strain path not only for the initial elastic part, but also for the final plastic part, where the elastic strains have a non-negligible influence on the slope of the strain paths.

Figure 10 also shows the influence of the parameter ω that controls the absolute rate of rotation of the yield surface toward its current target value (Equation (12)). For isotropic loading, the target value is α = 0, i.e., a non-inclined yield surface and null plastic deviatoric strains because an associated flow rule is used in the proposed model. Thus, for ω = 50, the yield surface rotates faster to the horizontal (hydrostatic) axis (Figure 1), deviatoric plastic strains are quickly 0 and the strain path quickly bends to the right again due to the positive deviatoric elastic strains. However, the slopes of the initial (elastic) part and the final (plastic) part are not the same because of the different values of the elastic and plastic volumetric strains.

## 6. Conclusions 

A novel constitutive model for soft structured clays that includes anisotropic behavior both of elastic and plastic nature has been developed. The new model incorporates stress-dependent cross-anisotropic elastic behavior within the yield surface using only an additional parameter to describe anisotropic elasticity, namely the square root of the ratio between horizontal and vertical stiffnesses.

Comparisons with laboratory results for Bothkennar clay show the procedure to analytically obtain the additional anisotropic elastic parameter from conventional triaxial tests and the improved capabilities of the presented model, namely the initial non-vertical effective stress paths for undrained triaxial tests and the non-null deviatoric strains during isotropic loading or unloading.

The proposed model clearly improves the predictive capabilities of its parent constitutive model (S-CLAY1S) at an element test level without notable complexity. Further validation of the model should be performed for boundary value problems.

## Figures and Tables

**Table 1:** Model parameters for Bothkennar clay.

κ	$\nu^{*}$	λ	M	ω	$\omega_{d}$	$e_{0}$	${\sigma^{\prime }}_{Vc}$ (kPa)	$\alpha_{0}$	$\alpha_{e}$
0.025	0.2	0.3	1.4	30	1	1.55 ^1^	90	0.3	1.3 ^2^

^1^ For a reference isotropic pressure of 15 kPa; ^2^ Using Equation (23) and triaxial data after McGinty [31].

**Table 2:** Strain path directions for triaxial tests at different strain ratios [31].

Test	C1	C2	C3	C4	C5	C6	C7	C9
η	0.42	1.11	1.30	−0.80	0.80	0.20	0.42	−0.50
$d\varepsilon_{d}^{e}/d\varepsilon_{v}^{e}$ ^1^	0.36	0.75	0.92	−0.65	0.53	0.2	0.53	−0.28
$d\varepsilon_{d}^{e}/d\varepsilon_{v}^{e}$ ^2^	0.25	0.38	0.47	−0.31	0.25	0.16	0.23	−0.06

^1^ Initial loading; ^2^ Unloading.

**Table 3:** Strain path directions for isotropic loading [31].

Test	B1	B2	B3	B4	B5	B6	B7	B8
η	0	0	0	0	0	0	0	0
$d\varepsilon_{d}^{e}/d\varepsilon_{v}^{e}$ ^1^	0.20	0.20	0.20	0.20	0.14	0.18	0.21	0.31
$d\varepsilon_{d}^{e}/d\varepsilon_{v}^{e}$ ^2^	0.08	0.14	0.18	0.20	0.15	0.10	0.12	0.07

^1^ Initial loading; ^2^ Unloading.

**Table 4:** Additional model parameters to include destructuration for Bothkennar clay.

$\lambda_{i}$	ξ	$\xi_{d}$	$\chi_{0}$
0.3	11	0.2	8
